# Key role of quorum‐sensing mutations in the development of *Staphylococcus aureus* clinical device‐associated infection

**DOI:** 10.1002/ctm2.801

**Published:** 2022-04-07

**Authors:** Lei He, Feiyang Zhang, Ying Jian, Huiying Lv, Musha Hamushan, Junlan Liu, Yao Liu, Hua Wang, Jin Tang, Pei Han, Dylan J. Burgin, Seth W. Dickey, Hao Shen, Min Li, Michael Otto

**Affiliations:** ^1^ Department of Laboratory Medicine Ren Ji Hospital Shanghai Jiao Tong University School of Medicine Shanghai China; ^2^ Department of Orthopedics Shanghai Jiao Tong University Affiliated Sixth People's Hospital Shanghai China; ^3^ Clinical Laboratory Department Shanghai Jiao Tong University Affiliated Sixth People's Hospital Shanghai China; ^4^ Pathogen Molecular Genetics Section Laboratory of Bacteriology National Institute of Allergy and Infectious Diseases US National Institutes of Health Bethesda Maryland USA; ^5^ Department of Orthopedics Jinjiang Municipal Hospital Fujian China; ^6^ Faculty of Medical Laboratory Science Shanghai Jiao Tong University School of Medicine Shanghai China

Dear Editor,

In the present study, we show that the development of *Staphylococcus aureus* device‐associated infection involves mutations in the quorum‐sensing system Agr that increase biofilm formation and thereby bacterial resistance to antibiotics.

The enormous difficulty clinicians face in the treatment of device‐associated infections is due to the characteristic involvement of biofilms, which are bacterial agglomerations that form on the device surface and exhibit considerably increased resistance to virtually all types of antibiotics.^1^ One of the most frequent causes of device‐associated infections is *Staphylococcus aureus*, which is also infamous for its exceptional recalcitrance towards antibiotic treatment.^2^, ^3^ How device‐associated infections develop remains incompletely understood. This is in part since only end‐point isolates from clinical device‐associated infection are usually investigated. Main questions that remain include whether a device‐associated infection is only caused by a selection of strains that are particularly pronounced biofilm formers or whether adaptations increasing biofilm formation occur during infection, and if so, which among them play a dominant role for that adaptation.

To answer these questions, we compared sequential isolates from cases of prolonged *S. aureus* surgical implant infection obtained at our hospital (See Figure S1 for selection of isolates). In all cases, initial (T1) samples were taken after infection had developed post‐surgery, and secondary (T2) samples were collected when recurrence or persistence of infection was diagnosed (Table S1). All bacterial samples from the T1 and T2 time points were classified as *S. aureus* by appearance and mass spectrometry. Remarkably, all T1 isolates were homogenously hemolytic on sheep blood agar plates, whereas all T2 samples were homogenously non‐hemolytic (Figure 1A). One representative clone of each sample was stored, and all stored T1 and T2 isolates were confirmed to exhibit a hemolytic and non‐hemolytic phenotype, respectively (Figure 1B). Notably, all T2 isolates also showed significantly higher in vitro biofilm‐forming capacities than the corresponding T1 isolates (Figure 1C).

**FIGURE 1:**
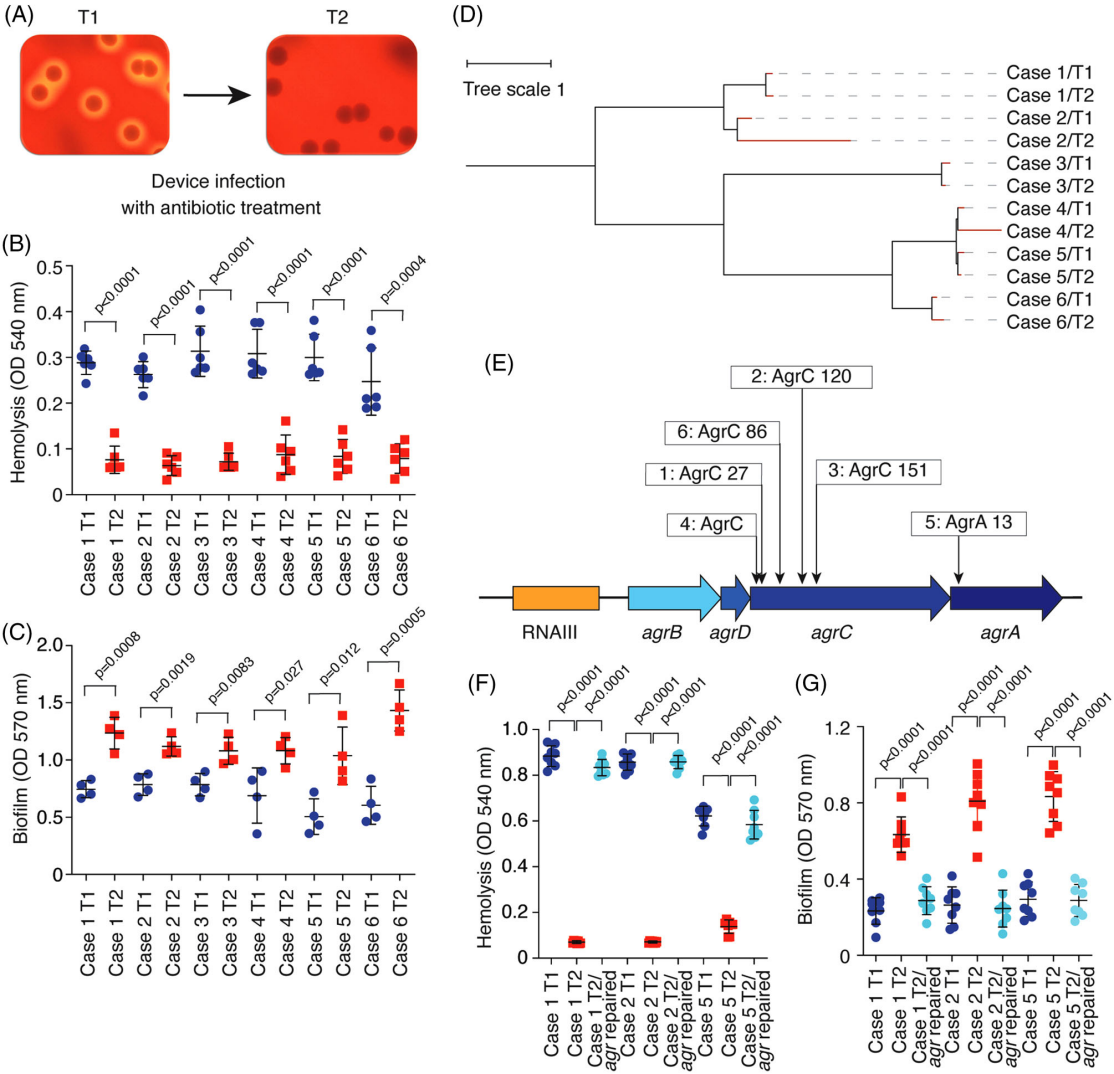
Biofilm‐increasing *agr* mutations develop during surgical implant infection. (A) Example of corresponding initial (T1) and secondary (T2) isolates analyzed on sheep blood agar plates. (B) Hemolytic capacities of T1 and T2 isolates from the six studied clinical cases. Hemolytic activities of bacterial culture filtrates from cultures grown for 15 h in tryptic soy broth were determined by incubation with human red blood cells for 1 h at 37°C and measuring the optical density at 540 nm. *n* = 6/group. (C) In vitro biofilm formation of the same isolates as in (B). Biofilms were grown in tryptic soy broth supplemented with .5% glucose for 48 h in 96‐well microtiter plates. Biofilm formation was measured using absorption at 570 nm after crystal violet staining. *n* = 4/group. (D) Relatedness of isolates. The tree was generated using the whole genome alignment 21.0 plugin to the QIAGEN CLC Genomics Workbench. It was constructed using the method of neighbor joining, which is well‐suited for trees with varying rates of evolution (since there are different ST types). The reference genome is ST398S0385. (E) Location of the non‐synonymous mutations in the *agr* locus detected in the T2 isolates. Numbers refer to amino acid positions in the encoded proteins. (F and G) Repair of *agr* mutations in T2 isolates. Hemolysis (F) and biofilm formation (G) of T1, T2 and repaired T2 isolates is shown. *n* = 8/group. (B and C) Statistical analysis is by two‐tailed, unpaired *t*‐tests. (F and G) Statistical analysis is by one‐way analysis of variance with Tukey's post‐tests. All error bars show the mean ± standard deviation (SD)

To analyze whether genetic changes were involved in the altered phenotypes that we observed, we performed whole genome sequencing (WGS). WGS confirmed that T2 isolates were derived from the T1 isolates, as they shared several characteristics and were closely genetically related, while the isolates from different cases were genetically distant (Figure 1D, Table S1). Only the locus encoding the Agr (accessory gene regulator) quorum‐sensing (QS) system^4^ showed non‐synonymous substitutions in all six isolates (Figure 1E, Tables 1 and 2, Table S2), indicating that the consistently observed phenotypes of reduced hemolysis and increased biofilm formation in the secondary isolates were due to Agr dysfunctionality. This idea is substantiated by the fact that non‐synonymous mutations in *agr*, but not in other genes with non‐synonymous single nucleotide polymorphism (SNPs) in our isolates, explain the hemolysis and biofilm phenotypes. This is because Agr‐dysfunctional mutants are known to be 1. non‐hemolytic, as Agr controls the production of secreted hemolysins,^4^ and 2. exhibit increased biofilm formation due to the absence of the strictly Agr‐controlled biofilm‐structuring phenol‐soluble modulin peptides and Agr‐dependent regulation of biofilm matrix‐degrading enzymes.^5^ Furthermore, the lack of hemolysis is often used in larger screens to detect Agr dysfunctionality, because it is almost always due to mutations in *agr*.^6^ The fact that all T2 samples were assessed as homogenously non‐hemolytic indicates that the Agr‐dysfunctional mutants that developed during device‐associated infection overtook virtually the entire bacterial device‐associated population.

**TABLE 1:** Isolate characteristics

**Isolate**	**Van** **^†^**	**Lev** **^†^**	**Cli** **^†^**	**Sequence type (ST)**	**MRSA/MSSA**	**SCCmec type (*ccr* allotype)**	**Agr subgrouptype**	**SNPs compared to T1**	***agr* mutation**
Case 1 – T1	1	.25	.6	ST398	MRSA	V (5C2)	IV		
Case 1 – T2	1	.25	.6	ST398	MRSA	V (5C2)	IV	112	*agrC* 83T insertion, frameshift
Case 2 – T1	1	.25	.6	ST398	MRSA	V (5C2)	IV		
Case 2 – T2	1	.25	.6	ST398	MRSA	V (5C2)	IV	123	*agrC* 358T deletion, frameshift
Case 3 – T1	1	.25	80^‡^	ST59	MRSA	IVa (2B)	I		
Case 3 – T2	1	.25	80^‡^	ST59	MRSA	IVa (2B)	I	111	*agrC* 453T insertion, frameshift
Case 4 – T1	1	1	.6	ST1281	MSSA		I		
Case 4 – T2	1	1	.6	ST1281	MSSA		I	90	*agrC* A58C (Met to Leu), TTAC62‐65ACTT (Phe to Tyr, Thr to Leu)
Case 5 – T1	.5	1	.6	ST1281	MSSA		I		
Case 5 – T2	.5	1	.6	ST1281	MSSA		I	122	*agrA* A37T (Arg to Stop)
Case 6 – T1	.5	1	.6	ST2631	MSSA		I		
Case 6 – T1	.5	1	.6	ST2631	MSSA		I	57	*agrC* C256T (Gln to Stop)
LAC	1	4	.3	ST8	MRSA	IVa (2B)	I		
LAC Δ*agr*	1	4	.3	ST8	MRSA	IVa (2B)	I		Entire deletion

Abbreviations: Cli, clindamycin; LAC, Los Angeles County clone; Lev, levofloxacin; MRSA/MSSA, methicillin‐resistant S. aureus/methicillin‐sensitive S. aureus; Van, vancomycin.

^†^μg ml^–1^.

^‡^considered resistant.

**TABLE 2:** Numbers of non‐synonymous substitutions per gene and case

**Gene**	**Case 1**	**Case 2**	**Case 3**	**Case 4**	**Case 5**	**Case 6**	**Number of cases with SNP in gene**
*agr locus*	1	1	1	3	1	1	6
*agrA*	0	0	0	0	1	0	1
*agrC*	1	1	1	3	0	1	5
*istA*	1	1	0	0	0	0	2
*xkdX*	1	0	0	0	0	0	1
*sdrD*	2	2	0	0	0	0	2
*coa*	1	0	0	0	0	0	1
*rnfC*	3	0	0	0	0	0	1
*arsR2*	2	0	0	0	0	0	1
*arsB*	1	0	0	0	0	0	1
*abgT*	1	0	0	0	0	0	1
*lpl*	0	1	0	0	0	0	1
*yhcN*	0	2	0	0	0	0	1
*clfB*	0	1	0	1	1	0	3
*cstA*	0	1	0	0	0	0	1
*sdrC*	0	0	1	0	0	0	1
*M013TW_1443*	0	0	1	0	0	0	1
*M013TW_1444*	0	0	1	0	0	0	1
*terL*	0	0	1	0	0	0	1
*M013TW_1441*	0	0	1	0	0	0	1
*M013TW_2107*	0	0	1	0	0	0	1
*sasG*	0	0	1	0	0	0	1
*arcC*	0	0	0	0	0	1	1

For the analysis of antibiotic resistance exhibited by the T1 and T2 isolate pairs, we chose levofloxacin (Lev), vancomycin (Van) and clindamycin (Cli) as they are in frequent clinical use for surgical implant infections and were among the most frequently used antibiotics in our study (Table S1). In addition, we included a strain pair consisting of a defined, isogenic *agr* deletion (Δ*agr*) mutant and the corresponding parental wild‐type of the most widespread clinical *S. aureus* type in the US, pulsed‐field type USA300 (Los Angeles County Clone, LAC).^7^ The minimal inhibitory concentration (MICs) for the tested antibiotics were unchanged between T1 and T2 isolates (Table 1). MIC measurements are performed using planktonic cells and cannot measure differences in antibiotic resistance that are due to variations in biofilm formation. When measured by survival assays in biofilm mode, all T2 isolates (with *agr* non‐synonymous substitutions) and the defined LACΔ*agr* mutant showed increased resistance to all tested antibiotics compared to the T1 isolates or the LAC wild‐type strain, respectively (Figure 2A). Confocal microscopy results confirmed that extended biofilm formation and increased resistance to all three tested antibiotics was associated with Agr dysfunctionality (Figure 2B; Figure S2). Finally, we selected three T2 isolates in which we repaired the detected *agr* mutation by an allelic replacement procedure. In all T2 isolates with a repaired *agr* sequence, biofilm and hemolysis phenotypes as well as antibiotic resistance in biofilm mode were restored to the levels of the T1 isolates (Figure 1F,G; Figure S3).

**FIGURE 2:**
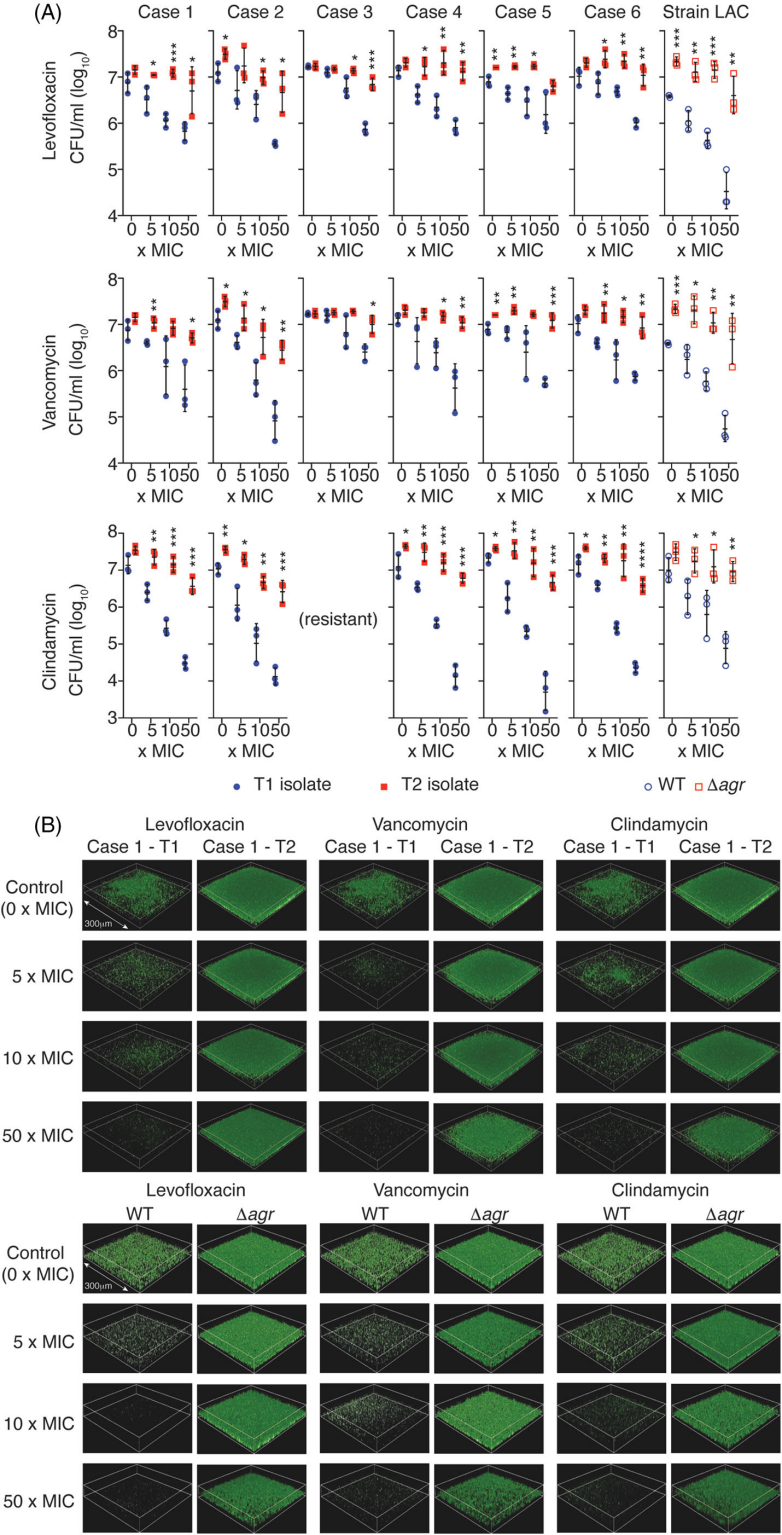
Biofilm antibiotic resistance of Agr‐functional and dysfunctional isolates. (A) Resistance to vancomycin, levofloxacin and clindamycin of sequential clinical isolates and LAC versus LACΔ*agr* isogenic strains in biofilm mode of growth. Biofilms were grown and measured as described in the legend to Figure 1. Concentrations are given as × MIC (measured in planktonic mode of growth using Clinical & Laboratory Standards Institute guidelines). See Table 1 for determined MICs for all isolates. Note case 3 isolates were not tested for biofilm‐mediated resistance to clindamycin as they were resistant to clindamycin as per their MIC. *n* = 3/group. Error bars show the geometric mean and geometric SD. *p < .05; **p < .01; ***p < .001; ****p < .0001 (unpaired two‐tailed *t*‐tests of corresponding T1/WT and T2/Δ*agr* values). (B) Analysis of biofilm formation and biofilm antibiotic resistance by CLSM for Case 1 T1 versus T2 isolates and LAC versus LACΔ*agr* isogenic strains. Biofilms were treated with antibiotic at the indicated concentrations for 24 h after the initial 48 h of biofilm growth. They were then stained with SYTO 9 (50 nM, 30 min) and imaged with an Operetta CLS high‐content imager (Perkin Elmer). The shown biofilm squares have a side length of 300 μm in every picture. See Figure S2 for Case 2 and Case 5 isolates

In conclusion, our results indicate that the formation of biofilms that is a hallmark of *S. aureus* device‐associated infection and leads to enhanced resistance to antibiotic treatment is a trait that can develop during clinical device‐associated infection by the development of non‐synonymous mutations in the Agr quorum‐sensing system. These findings have important clinical implications. Most notably, they suggest that antibiotic treatment that does not readily eradicate a device‐associated infection may be counterproductive as it can further exacerbate the infection by selecting for antibiotic‐resistant, biofilm‐forming mutants.

We previously showed increased capacity of Agr‐dysfunctional mutants to form biofilms.^8^ Agr‐dysfunctional mutants are also often observed among *S. aureus* infection isolates, particularly those obtained from blood infections, which frequently originate from device‐associated infections.^6^, ^9^ Nevertheless, the consistent involvement of Agr mutation in prolonged surgical implant infection we detected is astounding. Similarly remarkable was our finding indicating virtual homogeneity of Agr‐dysfunctional bacteria colonizing the T2 implants, which to a certain extent contradicts predictions from in vitro sociobiological models. Apparently, the selective pressures associated with biofilm‐mediated resistance of *agr* mutants, including resistance to antibiotics as shown here and to leukocyte attacks as we showed previously,^10^ significantly outweigh those favoring the maintenance of Agr‐functional bacteria during *S. aureus* device‐associated infection.

## CONFLICT OF INTEREST

The authors declare that they have no competing interests.

## Supporting information

Supporting Information

Supporting Information

Supporting Information

Supporting Information

Supporting Information
