# Supplementary figures and images for: Key role of quorum‐sensing mutations in the development of Staphylococcus aureus clinical device‐associated infection
Source: Clin Transl Med. 2022 Apr 7;12(4):e801. doi: 10.1002/ctm2.801 (PMC8989080; doi:10.1002/ctm2.801)

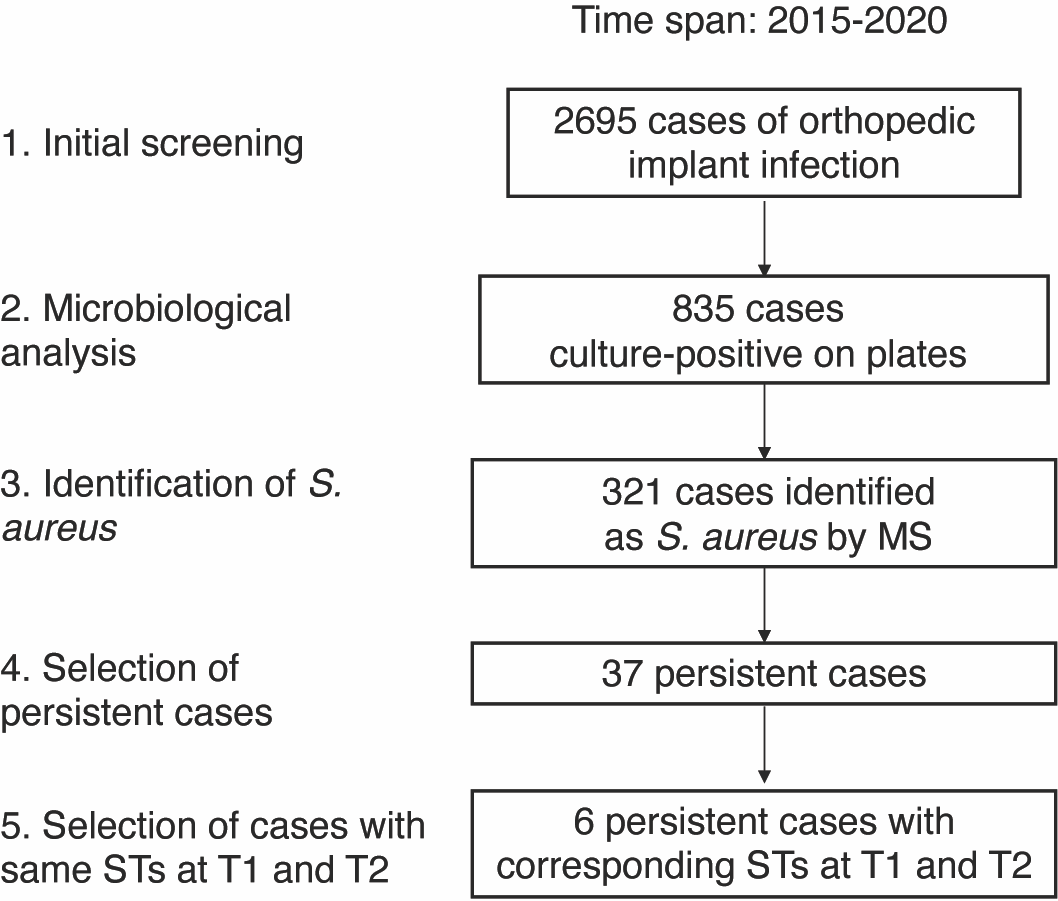


**Supporting Figure 1. Flowchart of isolate selection.**

Supplement: Supplementary file 1 — Supporting Information [file CTM2-12-e801-s002.docx]
