# Supplementary material for: Key role of quorum‐sensing mutations in the development of Staphylococcus aureus clinical device‐associated infection
Source: Clin Transl Med. 2022 Apr 7;12(4):e801. doi: 10.1002/ctm2.801 (PMC8989080; doi:10.1002/ctm2.801)
Supplement: Supplementary file 2 — Supporting Information [file CTM2-12-e801-s005.docx]

**
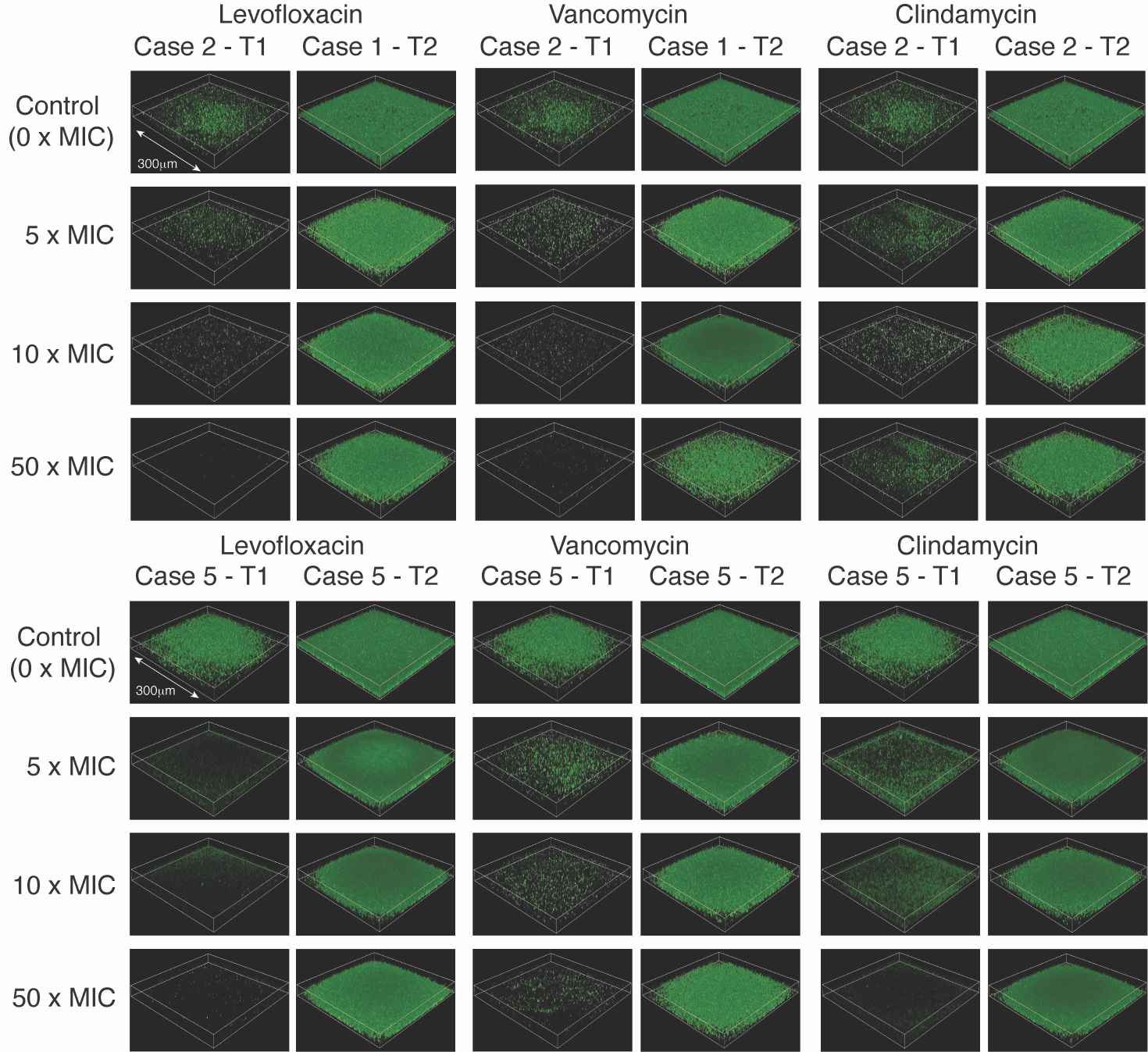
**

**Supporting Figure 2. Analysis of biofilm formation and biofilm antibiotic resistance by CLSM for Case 2 and 5 T1 versus T2 isolates.** See legend to **Fig. 2B** for technical details.
