# Supplementary material for: Key role of quorum‐sensing mutations in the development of Staphylococcus aureus clinical device‐associated infection
Source: Clin Transl Med. 2022 Apr 7;12(4):e801. doi: 10.1002/ctm2.801 (PMC8989080; doi:10.1002/ctm2.801)
Supplement: Supplementary file 3 — Supporting Information [file CTM2-12-e801-s004.docx]

**
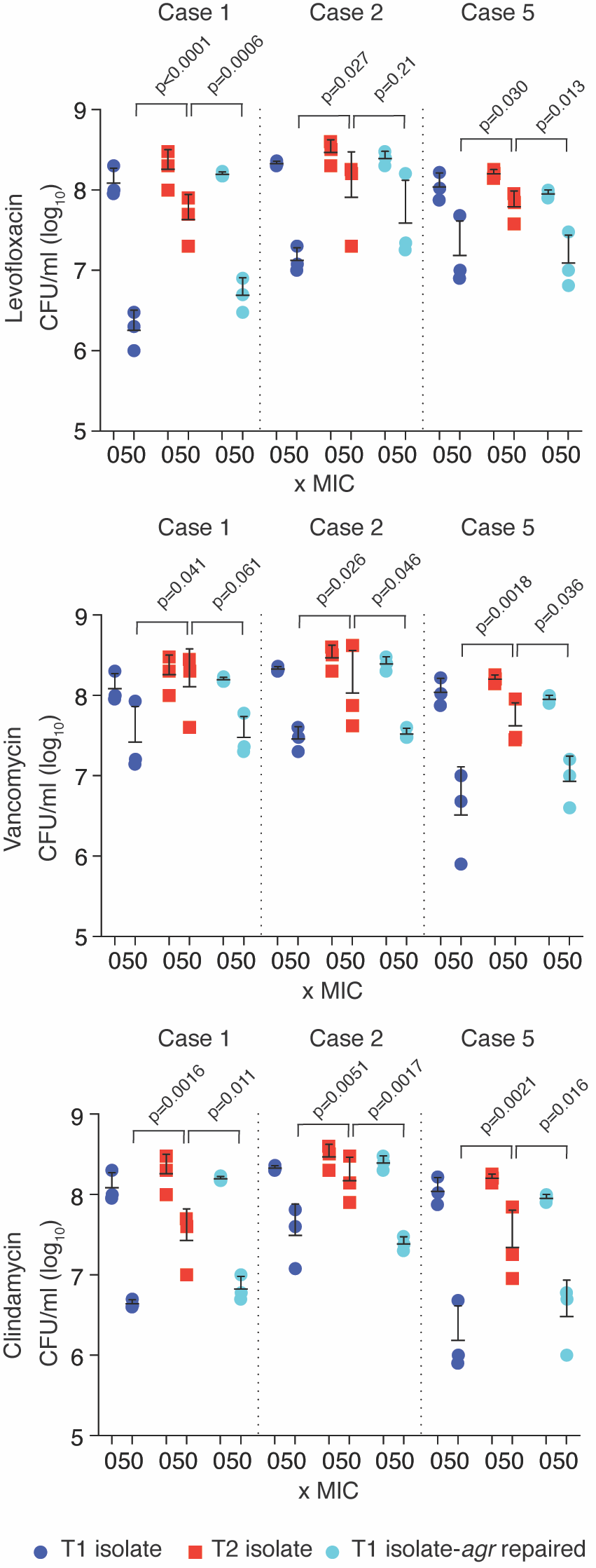
**

**Supporting Figure 3. Antibiotic resistance in biofilm mode of agr-repaired strains.** Analyses were performed without antibiotic and at 50 × MIC concentration. See legend to **Fig. 2A** for technical details. Statistical analysis is by 2-way ANOVAs with Tukey’s post-tests. n=3/group.
