# Supplementary material for: Key role of quorum‐sensing mutations in the development of Staphylococcus aureus clinical device‐associated infection
Source: Clin Transl Med. 2022 Apr 7;12(4):e801. doi: 10.1002/ctm2.801 (PMC8989080; doi:10.1002/ctm2.801)
Supplement: Supplementary file 4 — Supporting Information [file CTM2-12-e801-s001.pdf]

Supporting Table 1. Clinical case characteristics

Case number	Patient sex	Patient age	Diagnosis	Fixture	Surgery Date	Medication history	T1/T2 isolation Date
1	Male	58	Postoperative infection, right radius fracture	Plate, screw	2016/ June	2017/6/30-2017/7/10 Vancomycin 1g/q12h iv and Levofloxacin 0.5g/qd iv	T1 2017/6/26
						2017/10/23-2017/10/23 Levofloxacin 0.5g/qd iv and Gentamicin 80000u/q12h iv	T2 2017/10/24
2	Male	40	Postoperative infection, right ankle fracture	Plate, screw	2016/8/31	2016/9/14-16 Clindamycin 0.25g/q12h iv<br>2016/9/14-26 Levofloxacin 0.5g/qd iv	T1 2016/9/14
						2017/8/16-18 Levofloxacin 0.5g/qd iv	T2 2017/8/17
3	Male	36	Postoperative infection after, repair of left cruciate ligament	Screw	2017/7/28	2017/7/25-27 Clindamycin 0.25g/q12h iv<br>2017/9/13-18 Clindamycin 0.25g/q12h iv<br>2017/9/18-25 Levofloxacin 0.5g/qd iv	T1 2017/9/14
						2017/11/26-12/13 Levofloxacin 0.5g/qd iv<br>2017/11/27-30 Vancomycin 1g/q12h iv	T2 2017/11/29
4	Male	65	Postoperative infection, left foot fracture	Kirschner wire	2016/ February	2018/12/1-22 Cefotiam 0.5g/qd iv<br>2018/12/5-22 Levofloxacin 0.5g/qd iv	T1 2018/12/1
						2019/1/18-28 Cefotiam 2g/q12h iv<br>2019/1/30-02/03 Levofloxacin 0.5g/qd iv<br>2019/1/30-02/03 Ceftazidime 1.5g/q12h iv<br>2019/2/14-03/05 Moxifloxacin 0.4g/qd iv<br>2019/2/14-18 Vancomycin 1g/q12h iv	T2 2019/1/31
5	Male	76	Postoperative infection, right tibiofibular fracture	Plate, screw	2017/8/8	2017/6/10-13 Levofloxacin 0.5g/qd iv<br>2017/6/13-19 Moxifloxacin 0.4g/qd iv<br>2018/7/03-05 Levofloxacin 0.5g/qd iv<br>2018/7/05-11 Cefoperazone and Sulbactam 1.5g/q12h iv	T1 2018/7/4
						2019/4/12-17 Levofloxacin 0.5g/qd iv<br>2019/4/17-24 Vancomycin 1g/q12h iv	T2 2019/4/12
6	Male	32	Postoperative infection, right tibial fracture	Plate, screw	2014/ December	2017/12/21-23 Clindamycin 0.25g/q12h iv<br>2017/12/21-23 Levofloxacin 0.5g/qd iv	T1 2017/12/22
						2018/5/31-06/11 Aztreonam 1g/q12h iv	T2 2018/6/1
