# Supplementary material for: Key role of quorum‐sensing mutations in the development of Staphylococcus aureus clinical device‐associated infection
Source: Clin Transl Med. 2022 Apr 7;12(4):e801. doi: 10.1002/ctm2.801 (PMC8989080; doi:10.1002/ctm2.801)
Supplement: Supplementary file 5 — Supporting Information [file CTM2-12-e801-s003.pdf]

Supporting Table 2. Non-synonymous substitutions in sequential clinical isolates

Case	Location of mutation (gene)	Base change	Type of mutation	Amino acid exchange
Case 1	*istA*	C364T	Missense	Pro122Ser
	xkdX	T124A	Missense	Phe42Ile
	sdrD	3911_3912delinsGC	Deletion-insertion	Asp1304Gly
		C3596T	Missense	Ser1199Leu
	coa	G1773C	Missense	Lys591Asn
	rnfC	T452C	Missense	Val151Ala
		G403A	Missense	Ala135Thr
		C398T	Missense	Ala133Val
	arsR2	A4T	Missense	Thr2Ser
		T46C	Missense	Ser16Pro
	arsB	A303G	Missense	Ile101Met
	abgT	G1356A	Missense	Met452Ile
	agrC	83insT	Insertion	Frameshift
Case 2	*istA*	C364T	Missense	Pro122Ser
	sdrD	T3596C	Missense	Leu1199Ser
		G3857A	Missense	Gly1286Asp
	lpl	G1369A	Missense	Asp457Asn
	yhcN	C131A	Missense	Thr44Lys
		G290A	Missense	Ser97Asn
	agrC	358delT	Deletion	Frameshift
	clfB	C2260T	Missense	Leu754Phe
	cstA	A769G	Missense	Ile257Val
Case 3	*sdrC*	872insTAGCGA	Insertion	Frameshift
	M013TW_1443	675_676delinsGT	Deletion-insertion	Leu226Val
	M013TW_1444	663_664delinsTG	Deletion-insertion	Asn221_Asp222delinsLysTyr
	terL	1461_1462delinsCA	Deletion-insertion	Asn488Asp
	M013TW_1441	A244T	Missense	Thr82Ser
	agrC	453insT	Insertion	Frameshift
	M013TW_2107	G970A	Missense	Val324Ile
	sasG	A2789G	Missense	Lys930Arg
Case 4	*clfB*	1827_1831delATTCA	Deletion	Phe610frameshift
	agrC	A58C	Missense	Met20Leu
		62_63delinsAC	Deletion-insertion	Phe21Tyr
		64_65delinsTT	Deletion-insertion	Thr22Leu
Case 5	*clfB*	A1915C	Missense	Ile639Leu
	agrA	A37T	Missense	Arg13Stop
Case 6	*arcC*	94_95delAT	Deletion	Met32frameshift
	agrC	C256T	Missense	Ile23Phe
